# Static analysis of composite beams on variable stiffness elastic foundations by the Homotopy Analysis Method

**DOI:** 10.1007/s00707-021-03043-z

**Published:** 2021-08-13

**Authors:** Olga Doeva, Pedram Khaneh Masjedi, Paul M. Weaver

**Affiliations:** Bernal Institute, School of Engineering, University of Limerick, Limerick, Ireland

## Abstract

New analytical solutions for the static deflection of anisotropic composite beams resting on variable stiffness elastic foundations are obtained by the means of the Homotopy Analysis Method (HAM). The method provides a closed-form series solution for the problem described by a non-homogeneous system of coupled ordinary differential equations with constant coefficients and one variable coefficient reflecting variable stiffness elastic foundation. Analytical solutions are obtained based on two different algorithms, namely conventional HAM and iterative HAM (iHAM). To investigate the computational efficiency and convergence of HAM solutions, the preliminary studies are performed for a composite beam without elastic foundation under the action of transverse uniformly distributed loads considering three different types of stacking sequence which provide different levels and types of anisotropy. It is shown that applying the iterative approach results in better convergence of the solution compared with conventional HAM for the same level of accuracy. Then, analytical solutions are developed for composite beams on elastic foundations. New analytical results based on HAM are presented for the static deflection of composite beams resting on variable stiffness elastic foundations. Results are compared to those reported in the literature and those obtained by the Chebyshev Collocation Method in order to verify the validity and accuracy of the method. Numerical experiments reveal the accuracy and efficiency of the Homotopy Analysis Method in static beam problems.

## Introduction

Laminated composite structures resting on elastic foundations are increasingly used in aerospace, marine, civil, biomedical, and other engineering applications due to their high strength-to-weight ratio, improved damage tolerance nature, and corrosion resistance. Composite beams are fundamental structural elements, and understanding of their static deflection behaviour is essential for engineering design and modelling purposes. Analytical solutions offer suitable tools to assist in this process and provide an insight into the governing physics of the problem allowing critical characteristics of composite structures on variable elastic foundations to be analysed and predicted. Different analytical approaches have been applied to analyse static and dynamic structural behaviour of beams resting on elastic foundations, for example, solutions such as that following Navier [1–7], Fourier series [8–12], Power series [13–15], Variational Iteration Method [16–20], and Homotopy Perturbation Method [19, 21–23]. However, these techniques suffer from significant drawbacks. For example, Navier’s solution and Fourier series methods, in which all variables and their derivatives are expressed by trigonometric series expansions, are limited to specific simply supported boundary conditions. When applied to other boundary conditions, Fourier series tends to become slowly convergent, and its derivatives are not easily obtained and could even be discontinuous. These limitations can be overcome by modified Fourier series enriched with auxiliary polynomials noting that obtaining them poses additional difficulties. A power series solution involves discretisation of variables which usually requires large amounts of computer memory and processing time, leading to rounding errors, and thus results in loss of accuracy. The Variational Iteration Method requires finding the general Lagrange multiplier which is a challenging task in complex problems. Results provided by the Homotopy Perturbation Method highly depend on the good choice of a linear operator and initial guess. The Homotopy Analysis Method was proposed by Liao in [24] and further developed in [25] as an efficient analytical technique for solving nonlinear differential equations. The method employs the concept of the homotopy in topology and generates a solution in a form of convergent series. The main tenet of HAM is to replace a nonlinear equation by a system of linear ordinary differential equations that can be solved easily with the help of symbolic software packages such as MATLAB or Maple. In contrast to previously mentioned methodologies, HAM is an easy-to-use analytical tool which is general in terms of loading or boundary conditions. It does not involve discretisation or any physical parameters, small or large. Unlike other analytical approaches, HAM allows the convergence region and rate of convergence to be controlled by using an auxiliary parameter. Flexibility in the choice of this auxiliary parameter as well as linear operator and initial guess is another great advantage of the method. The Homotopy Analysis Method has been successfully applied to solve many problems in biology [26–28], chemistry [29–31], physics [32–34], optimal control theory [35–37], fluid mechanics [38–41], and solid mechanics [42–44]. More specifically, HAM successfully solved static and dynamic problems of isotropic beams. Wang et al. [45] used HAM to solve the bending problem of a cantilever beam under tip load resulting in large deformation. An accurate analytical expression for the rotation angle at the free end was obtained and then used to calculate the vertical and horizontal displacements of the beam. Kimiaeifar et al. [46] exploited HAM to solve analytically the large deflection and rotation of a nonlinear cantilever beam under the action of a co-planar follower static load. HAM results were compared with those obtained from a fourth-order Runge–Kutta method to prove the efficiency of the method. Later, Kimiaeifar et al. [47] used HAM to obtain an approximate solution for the governing equation of large deflection and rotation of a nonlinear Euler–Bernoulli beam with variable flexural rigidity loaded by circulatory forces. Maleki et al. [48] investigated the nonlinear large deformation of Euler–Bernoulli beams subject to arbitrary distributed loads by means of HAM. Deflection, slope, and bending moment were obtained for the case of constant and periodic distributed loads. Using HAM, Kimiaeifar et al. [49] derived an analytical solution for the large deflection analysis of an isotropic cantilever beam under the action of end point and uniformly distributed loads. Comparison of results with those obtained from the Finite Element Method (FEM) demonstrated the accuracy and computational efficiency of HAM. Liao [50] employed HAM to obtain convergent series solutions to eigenvalues and eigenfunctions for buckling of Euler–Bernoulli beams with uniform cross section under the action of axial load. Using the Galerkin method, Pirbodaghi et al. [51] transformed a nonlinear partial differential equation in space and time into an ordinary differential equation and then applied HAM to obtain analytical expressions for nonlinear vibration of Euler–Bernoulli beams subject to axial loads. By comparing HAM results against others in the literature, the accuracy of the method was demonstrated for simply supported and clamped beams. Hoseini et al. [52] employed HAM to obtain an accurate analytical solution for the fundamental nonlinear natural frequency and corresponding displacement of tapered beams presented by nonlinear differential equations of second order. Sedighi et al. [53] used HAM to derive analytical solutions for transversal vibration of hinged–hinged flexible beams subject to a constant load at their tips described by a fifth-order nonlinear differential equation. Excellent accuracy of HAM results was confirmed by comparing them against Runge–Kutta methods.

In the context of composite beams, Jafari-Talookolaei et al. [54] applied HAM to derive analytical expressions for the large amplitude free vibration of an unsymmetrically laminated composite beam on an elastic foundation under the action of axial load. An elastic foundation with quadratic and cubic nonlinearities was considered. Recently, Tang et al. [55] used HAM to find a closed-form solution for nonlinear free vibration of Euler–Bernoulli beams made of bi-directional functionally graded materials expressed by a third-order nonlinear partial differential equation which was transformed to ordinary differential equations (ODE) by means of the Galerkin method. The accuracy of the analytical results was confirmed by comparing them with those obtained by the Runge–Kutta method. Lin et al. [56] employed HAM to derive analytical expressions for the large deformation of axially functionally graded cantilever beams subject to tip loads. Results were compared with those from FEM to verify efficiency and accuracy.

Despite its advantages, HAM is a relatively new analytical approach in the context of composite structures, and the full potential of this method is not well known for solving various problems arising in this field. Mathematically, the coupled deflection of composite beams is a boundary value problem presented by a linear system of four coupled governing differential equations, reflecting four degrees of freedom, namely bending in two principal directions, axial elongation and twist [57–60]. In [61], this model was extended to composite beams resting on constant stiffness elastic foundations. A corresponding exact analytical solution was provided. In the current work, the model is further elaborated to composite beams resting on variable stiffness Winkler elastic foundations presented by a system of four coupled ordinary differential equations one of which has variable coefficients, noting that there is no exact solution for this newly developed problem. Thus, the main purpose of this work is to apply HAM, as a fresh attempt, to obtain a converged series solution for this problem and to investigate its accuracy, efficiency as well as factors affecting its convergence. To implement HAM, two different algorithms are used, one based on the traditional approach proposed in [24] and the other based on an iterative approach [62]. The accuracy and computational efficiency of both algorithms are evaluated by comparing their convergence and computational time required to obtain the results of the prescribed level of accuracy. In addition, to demonstrate the potential of HAM, results obtained from conventional and iterative approaches are compared with those obtained by the Chebyshev Collocation Method (CCM), which has proved to be very effective in solving beam problems [63–66].

The rest of the paper is organised as follows. In Sect. 2, a mathematical formulation of static deflection of composite beams resting on elastic foundations is given. In Sect. 3, a brief outline of the Homotopy Analysis Method is presented. In Sect. 4, the implementation of the method to the particular problem is explained. In Sect. 5, the validity and efficiency of the method are illustrated with some examples. A brief analysis of the obtained results is given. Finally, some discussions and conclusions are presented in Sect. 6.

## Problem statement

**Fig. 1:**
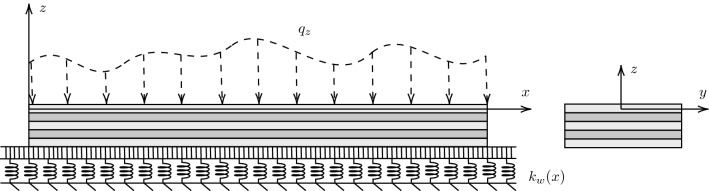
Composite beam on elastic foundation

Figure 1 illustrates a slender composite beam of length $\ell$ measured along the *x* coordinate axis with the cross section in the $y-z$ plane resting on a Winkler elastic foundation described by a set of mutually independent spring elements with modulus $k_w$. Due to imperfections and material non-homogeneity of the foundation, the elastic coefficient of the Winkler foundation can vary along the beam span, i.e. $k_w = k_w(x)$. The *x*-axis is assumed to be along the beam reference line.

Displacement of a generic point on the cross-section can be expressed as

1.1 $$\begin{aligned} U_x&= u(x) + z \theta _{y}(x) - y \theta _{z}(x), \end{aligned}$$

1.2 $$\begin{aligned} U_y&= v(x) - z \varphi (x), \end{aligned}$$

1.3 $$\begin{aligned} U_z&= w(x) + y \varphi (x), \end{aligned}$$

where $U_{x}$, $U_{y}$, and $U_{z}$ are the components of displacement vector; *u*, *v*, and *w* represent displacements of the beam reference line in *x*, *y*, and *z* directions; and $\varphi$, $\theta _{y}$, and $\theta _{z}$ are the rotations of the beam cross-section about *x*, *y*, and *z* axes, respectively.

Using Eq. (1.1), the strain measures of the beam can be written as

2.1 $$\begin{aligned} \epsilon _{xx} = \frac{\partial U_{x}}{\partial x} = u' + z \theta _{y}' - y \theta _{z}', \end{aligned}$$

2.2 $$\begin{aligned} \gamma _{xy} = \frac{\partial U_{x}}{\partial y} + \frac{\partial U_{y}}{\partial x} = \left( v'-\theta _{z}\right) - z \varphi ', \end{aligned}$$

2.3 $$\begin{aligned} \gamma _{xz} = \frac{\partial U_{x}}{\partial z} + \frac{\partial U_{z}}{\partial x} = \left( w'+\theta _{y}\right) + y \varphi '. \end{aligned}$$

According to Euler–Bernoulli beam theory, the cross-section of the beam remains orthogonal to the reference axis after deformation; therefore,

3 $$\begin{aligned} \theta _{y}=-w' , \quad \theta _{z}=v'. \end{aligned}$$

The internal work of the beam can be obtained by

4 $$\begin{aligned} \int _{V} W_{int} dV = \int _{V} \left( \sigma _{xx} \epsilon _{xx} + \sigma _{xy} \gamma _{xy} + \sigma _{xz} \gamma _{xz} \right) dV, \end{aligned}$$

where $\sigma _{xy}$, $\sigma _{xz}$, and $\sigma _{xx}$, are stresses in *x*, *y*, and *z* directions, respectively. Using definitions of the strain measures $\epsilon _{xx}$, $\gamma _{xy}$, and $\gamma _{xz}$ given by Eqs. (2.1), Eq. (4) can be rewritten as

5 $$\begin{aligned} \int _{V} W_{int} dV = \int _{V} \left( \sigma _{xx} u' + z \sigma _{xx} \theta _{y}' - y \sigma _{xx} \theta _{z}' + \sigma _{xy} \left( v'-\theta _{z} \right) + \sigma _{xz} \left( w'+\theta _{y}\right) + \left( y \sigma _{xz} - z \sigma _{xy} \right) \varphi ' \right) dV. \end{aligned}$$

Internal forces and moments of a beam can be expressed as

6.1 $$\begin{aligned} F_{x} = \int _{A} \sigma _{xx} dA, \end{aligned}$$

6.2 $$\begin{aligned} M_{x} = \int _{A} \left( y \sigma _{xz} - z \sigma _{xy} \right) dA, \end{aligned}$$

6.3 $$\begin{aligned} M_{y} = \int _{A} z \sigma _{xx} dA, \end{aligned}$$

6.4 $$\begin{aligned} M_{z} = -\int _{A} y \sigma _{xx} dA. \end{aligned}$$

Considering Eqs. (3) and (6.1), Eq. (5) can be further modified as follows

7 $$\begin{aligned} \int _{0}^{\ell } W_{int} dx = \int _{0}^{\ell } \left( F_{x} u' + M_{x} \varphi ' + M_{y} \theta _{y}' + M_{z} \theta _{z}' \right) dx = \int _{0}^{\ell } \left( F_{x} u' + M_{x} \varphi ' - M_{y} w'' + M_{z} v'' \right) dx. \end{aligned}$$

The principle of virtual work for a beam resting on an elastic foundation is written as

8 $$\begin{aligned} \int _{0}^{\ell } \left( \delta W_{int} + \delta W_f - \delta W_{ext} \right) dx = 0, \end{aligned}$$

where $\delta W_{int}$, $\delta W_f$, and $\delta W_{ext}$ are the variations in internal, elastic foundation, and external works, respectively. The variation in internal work of the beam can be obtained from Eq. (7) as follows:

9 $$\begin{aligned} \int _{0}^{\ell } \delta W_{int} = \int _{0}^{\ell } \delta \varvec{\varepsilon }^{T} {\varvec{N}} dx = \int _{0}^{\ell } \delta \varvec{\varepsilon }^{T}{\varvec{S}}\varvec{\varepsilon } dx, \end{aligned}$$

where vector of strains and curvatures $\varvec{\varepsilon }$, vector of internal forces and moments ${\varvec{N}}$, and stiffness matrix ${\varvec{S}}$ can be defined as

10 $$\begin{aligned} \varvec{\varepsilon } = \begin{bmatrix} \epsilon _{x} \\ \kappa _{x} \\ \kappa _{y} \\ \kappa _{z} \end{bmatrix} = \begin{bmatrix} u' \\ \varphi ' \\ -w'' \\ v'' \end{bmatrix}, \quad \quad {\varvec{N}} = \begin{bmatrix} F_{x} \\ M_{x} \\ M_{y} \\ M_{z} \end{bmatrix}, \quad \quad {\varvec{S}} = \begin{bmatrix} EA &{}\quad S_{ET} &{} \quad S_{EF} &{}\quad S_{EL} \\ S_{ET} &{}\quad GJ &{} \quad S_{FT} &{}\quad S_{LT} \\ S_{EF} &{} \quad S_{FT} &{} \quad EI_{y} &{}\quad S_{FL} \\ S_{EL} &{}\quad S_{LT} &{} \quad S_{FL} &{}\quad EI_{z} \end{bmatrix}, \end{aligned}$$

where $\epsilon _{x}$ is a strain, $\kappa _{x}$, $\kappa _{y}$, and $\kappa _{z}$ are curvatures in *x*, *y*, and *z* directions, respectively, *EA* is the extensional stiffness, *GJ* is the twist stiffness, $EI_y$ is the out-of-plane bending stiffness, $EI_z$ is the in-plane bending stiffness, $S_{ET}$ is the coupling between axial elongation and twist, $S_{EF}$ is the coupling between out-of-plane bending and axial elongation, $S_{EL}$ is the coupling between in-plane bending and axial elongation, $S_{FT}$ is the coupling between out-of-plane bending and twist, $S_{LT}$ is the coupling between in-plane bending and twist, and $S_{FL}$ is the coupling between out-of-plane and in-plane bending. It is worth mentioning that in Eq. (9) a linear relation between internal forces and moments ${\varvec{N}}$ and strains and curvatures $\varvec{\varepsilon }$ is assumed, i.e. ${\varvec{N}} = {\varvec{S}}\varvec{\varepsilon }$. For more details of this assumption, see [60].

The variation of external work is presented by

11 $$\begin{aligned} \int _{0}^{\ell } \delta W_{ext} \, dx = \int _{0}^{\ell } \delta \varvec{\overline{U}}^T {\varvec{Q}} dx, \end{aligned}$$

where the vector of displacements and rotations $\varvec{\overline{U}}$ and the vector of external forces and moments ${\varvec{Q}}$ are expressed by

12 $$\begin{aligned} \varvec{\overline{U}} = \begin{bmatrix} u \\ \varphi \\ w \\ v \end{bmatrix}, \quad \quad {\varvec{Q}} = \begin{bmatrix} q_{x} \\ q_{\varphi } \\ q_{z} \\ q_{y} \end{bmatrix}, \end{aligned}$$

where $q_{x}$, $q_{y}$, $q_{z}$ are functions of *x* representing distributed loads and $q_{\varphi }$ is the function of *x* representing distributed torque. Further, the variation of work due to the Winkler elastic foundation can be written as [3]

13 $$\begin{aligned} \int _0^{\ell } \delta W_{f} = \int _0^{\ell } \delta w \, k_w(x) \, w \, dx. \end{aligned}$$

Substituting Eqs. (9), (11), and (13) into Eq. (8), applying the integration by parts and collecting the coefficients of $\delta u$, $\delta \varphi$, $\delta w$, $\delta v$, $\delta w'$, and $\delta v'$, the set of governing equations can be obtained:

14 $$\begin{aligned} \begin{aligned} -&EA u'' - S_{ET} \varphi '' + S_{EF} w''' - S_{EL} v''' = q_x, \\ -&S_{ET} u'' - GJ \varphi '' + S_{FT} w''' - S_{LT} v''' = q_{\varphi }, \\ -&S_{EF} u''' - S_{FT} \varphi ''' + EI_{y} w^{(IV)} - S_{FL} v^{(IV)} + k_w(x) w = q_z, \\&S_{EL} u''' + S_{LT} \varphi ''' - S_{FL} w^{(IV)} + EI_{z} v^{(IV)} = q_y. \end{aligned} \end{aligned}$$

At $x=0$ and $x=\ell$, the boundary conditions can be expressed as follows

15.1 $$\begin{aligned} u&= 0, \end{aligned}$$

15.2 $$\begin{aligned} \varphi&= 0, \end{aligned}$$

15.3 $$\begin{aligned} w&= 0, \end{aligned}$$

15.4 $$\begin{aligned} v&= 0, \end{aligned}$$

15.5 $$\begin{aligned} w'&= 0, \end{aligned}$$

15.6 $$\begin{aligned} v'&= 0, \end{aligned}$$

16.1 $$\begin{aligned} EA u' + S_{ET} \varphi ' - S_{EF} w'' + S_{EL} v''&= 0, \end{aligned}$$

16.2 $$\begin{aligned} S_{ET} u' + GJ \varphi ' - S_{FT} w'' + S_{LT} v''&= 0, \end{aligned}$$

16.3 $$\begin{aligned} - S_{EF} u' - S_{FT} \varphi ' + EI_{y} w'' - S_{FL} v''&= 0, \end{aligned}$$

16.4 $$\begin{aligned} S_{EL} u' + S_{LT} \varphi ' - S_{FL} w'' + EI_{z} v''&= 0, \end{aligned}$$

16.5 $$\begin{aligned} S_{EF} u'' + S_{FT} \varphi '' - EI_{y} w''' + S_{FL} v'''&= 0, \end{aligned}$$

16.6 $$\begin{aligned} - S_{EL} u'' - S_{LT} \varphi '' + S_{FL} w''' - EI_{z} v'''&= 0, \end{aligned}$$

where $()'$ denotes the derivative with respect to *x*. The distribution of the variable stiffness Winkler foundation along the axial direction is assumed to be linear and varies according to the following rule:

17 $$\begin{aligned} k_w(x) = k_0 (1 - a x), \end{aligned}$$

where $k_0$ is the stiffness of the foundation, $1 - a x$ is a function of the spatial coordinate along the beam length, and *a* is a variation parameter of the elastic foundation.

## An overview of the Homotopy Analysis Method

Consider the differential equation

18 $$\begin{aligned} {\mathscr {N}} [u(x)] = 0, \end{aligned}$$

where ${\mathscr {N}}$ is a nonlinear operator, *x* is an independent variable, and *u*(*x*) is an unknown function. According to Liao [25], the zeroth-order deformation equation can be written as

19 $$\begin{aligned} (1 - q) {\mathscr {L}} [\phi (x;q) - u_0(x)] = q \hbar {\mathscr {N}} [\phi (x;q)], \end{aligned}$$

where $q \in [0, 1]$ is an embedding parameter, ${\mathscr {L}}$ is an auxiliary linear operator, $\phi (x;q)$ is the unknown function, $u_0(x)$ is the initial approximation of *u*(*x*), and $\hbar \ne 0$ is an auxiliary convergence control parameter. Setting the embedding parameter $q = 0$ leads to

20 $$\begin{aligned} \phi (x;0) = u_0(x), \end{aligned}$$

and when $q = 1$, Eq. (19) is equal to the original governing equation

21 $$\begin{aligned} \phi (x;1) = u(x), \end{aligned}$$

meaning that as *q* increases from 0 to 1, the solution $\phi (x;q)$ varies from the initial guess $u_0(x)$ to the solution *u*(*x*). Expanding $\phi (x;q)$ in a Maclaurin series with respect to *q* and using Eq. (20) leads to

22 $$\begin{aligned} \phi (x;q) = u_0(x) + \sum _{n=1}^{\infty } u_n(x) q^n, \end{aligned}$$

where

23 $$\begin{aligned} u_n(x) = \left. \frac{1}{n!} \frac{\partial ^n \phi (x;q)}{\partial q^n} \right| _{q=0}. \end{aligned}$$

HAM provides great freedom in choosing the auxiliary parameter $\hbar$. For an appropriately chosen value of $\hbar$, the series presented by Eq. (22) converges when $q = 1$, and thus,

24 $$\begin{aligned} u(x) = \phi (x;1) = u_0(x) + \sum _{n=1}^{\infty } u_n(x), \end{aligned}$$

where the unknown functions $u_n(x)$ can be obtained by using the *n*th-order deformation equation

25 $$\begin{aligned} {\mathscr {L}} [u_n(x) - \chi _n u_{n-1}(x)] = \hbar R_n(\mathbf {u}_{n-1}), \end{aligned}$$

where

26 $$\begin{aligned} \mathbf {u}(x) = \{ u_0(x), u_1(x), u_2(x), \ldots , u_n(x) \}, \end{aligned}$$

27 $$\begin{aligned} R_n(\mathbf {u}_{n-1}) = \left. \frac{1}{(n-1)!} \frac{\partial ^{n-1} {\mathscr {N}} [\phi (x;q)] }{\partial q^{n-1} } \right| _{q=0}, \end{aligned}$$

and

28 $$\begin{aligned} \chi _n = {\left\{ \begin{array}{ll} 0 &{}\text{ if } n \le 1, \\ 1 &{} \text{ if } n > 1. \end{array}\right. } \end{aligned}$$

The *n*th-order deformation equation (25) is derived by differentiating the zeroth-order deformation equation (19) *n* times with respect to *q*, dividing by *n*! and then setting $q = 0$. Finally, applying the inverse operator ${\mathscr {L}}^{-1}$, the expression for the unknown function can be obtained as follows:

29 $$\begin{aligned} u_n(x) = \chi _n u_{n-1}(x) + \hbar {\mathscr {L}}^{-1} [R_n(\mathbf {u}_{n-1})]. \end{aligned}$$

Substituting Eq. (29) into Eq. (24) leads to a series solution of Eq. (18).

## Implementation of the Homotopy Analysis Method

In order to solve the static deflection of the composite beam described by the system of coupled Eq. (14) by means of HAM, consider the auxiliary linear operators of the form

30.1 $$\begin{aligned} {\mathscr {L}}_u [\phi _u(x;q)]&= \frac{\partial ^2 \phi _u(x;q)}{\partial x^2}, \end{aligned}$$

30.2 $$\begin{aligned} {\mathscr {L}}_{\varphi } [\phi _{\varphi }(x;q)]&= \frac{\partial ^2 \phi _{\varphi }(x;q)}{\partial x^2}, \end{aligned}$$

30.3 $$\begin{aligned} {\mathscr {L}}_w [\phi _w(x;q)]&= \frac{\partial ^4 \phi _w(x;q)}{\partial x^4}, \end{aligned}$$

30.4 $$\begin{aligned} {\mathscr {L}}_v [\phi _v(x;q)]&= \frac{\partial ^4 \phi _v(x;q)}{\partial x^4}. \end{aligned}$$

This selection is based on the method of highest order differential matching which implies the use of the highest order derivative appearing in the differential equation. In turn, inverse operators are integral operators given by

31.1 $$\begin{aligned} {\mathscr {L}}_u^{-1}[(\cdot )]&= \iint (\cdot ) + c_1 x + c_2, \end{aligned}$$

31.2 $$\begin{aligned} {\mathscr {L}}_{\varphi }^{-1}[(\cdot )]&= \iint (\cdot ) + c_3 x + c_4, \end{aligned}$$

31.3 $$\begin{aligned} {\mathscr {L}}_w^{-1}[(\cdot )]&= \iiiint (\cdot ) + c_5 x^3 + c_6 x^2 + c_7 x + c_8, \end{aligned}$$

31.4 $$\begin{aligned} {\mathscr {L}}_v^{-1}[(\cdot )]&= \iiiint (\cdot ) + c_9 x^3 + c_{10} x^2 + c_{11} x + c_{12}, \end{aligned}$$

where $c_i$, $i = 1, 2, \ldots , 12$, are arbitrary constants of integration to be determined from boundary conditions. Defining nonlinear operators as

32.1 $$\begin{aligned} {\mathscr {N}}_u&= \frac{\partial ^2 \phi _u}{\partial x^2} + \frac{S_{ET}}{EA} \frac{\partial ^2 \phi _{\varphi }}{\partial x^2} - \frac{S_{EF}}{EA} \frac{\partial ^3 \phi _w}{\partial x^3} + \frac{S_{EL}}{EA} \frac{\partial ^3 \phi _v}{\partial x^3} - \frac{q_x}{EA}, \end{aligned}$$

32.2 $$\begin{aligned} {\mathscr {N}}_{\varphi }&= \frac{\partial ^2 \phi _{\varphi }}{\partial x^2} + \frac{S_{ET}}{GJ} \frac{\partial ^2 \phi _u}{\partial x^2} - \frac{S_{FT}}{GJ} \frac{\partial ^3 \phi _w}{\partial x^3} + \frac{S_{LT}}{GJ} \frac{\partial ^3 \phi _v}{\partial x^3} - \frac{q_{\varphi }}{GJ}, \end{aligned}$$

32.3 $$\begin{aligned} {\mathscr {N}}_w&= \frac{\partial ^4 \phi _w}{\partial x^4} - \frac{S_{EF}}{EI_y} \frac{\partial ^3 \phi _u}{\partial x^3} - \frac{S_{FT}}{EI_y} \frac{\partial ^3 \phi _{\varphi }}{\partial x^3} - \frac{S_{FL}}{EI_y} \frac{\partial ^4 \phi _v}{\partial x^4} + \frac{k_w(x)}{EI_y} \phi _w - \frac{q_z}{EI_y}, \end{aligned}$$

32.4 $$\begin{aligned} {\mathscr {N}}_v&= \frac{\partial ^4 \phi _v}{\partial x^4} + \frac{S_{EL}}{EI_z} \frac{\partial ^3 \phi _u}{\partial x^3} + \frac{S_{LT}}{EI_z} \frac{\partial ^3 \phi _{\varphi }}{\partial x^3} - \frac{S_{FL}}{EI_z} \frac{\partial ^4 \phi _w}{\partial x^4} - \frac{q_y}{EI_z}, \end{aligned}$$

*n*th-order deformation equations for the problem can be written as follows:

33.1 $$\begin{aligned} u_n(x)&= \chi _n u_{n-1}(x) + \hbar {\mathscr {L}}_u^{-1} [R_{u, n}(\mathbf {u}_{n-1})], \end{aligned}$$

33.2 $$\begin{aligned} \varphi _n(x)&= \chi _n \varphi _{n-1}(x) + \hbar {\mathscr {L}}_{\varphi }^{-1} [R_{{\varphi }, n}(\mathbf {\varphi }_{n-1})], \end{aligned}$$

33.3 $$\begin{aligned} w_n(x)&= \chi _n w_{n-1}(x) + \hbar {\mathscr {L}}_w^{-1} [R_{w, n}(\mathbf {w}_{n-1})], \end{aligned}$$

33.4 $$\begin{aligned} v_n(x)&= \chi _n v_{n-1}(x) + \hbar {\mathscr {L}}_v^{-1} [R_{v, n}(\mathbf {v}_{n-1})]. \end{aligned}$$

The next important step in employing the Homotopy Analysis Method is to choose the initial solution. Appropriate selection of the initial approximation (i.e. as close to the exact solution as possible) results in better accuracy and convergence of the approximate solution. A suitable choice for the static deflection problem of composite beams is to use the solution of uncoupled equations of isotropic beams as for different types of boundary equations these solutions are expressed by polynomial functions (as discussed in Sect. 5). The polynomial functions are easy to integrate, which in turn simplifies the process of deriving the *n*th-order deformation equations and reduces the computational time required to obtain the approximate solutions. However, as the order of deformation equation increases, the computational complexity grows rapidly. To overcome this issue and to increase the convergence and accuracy of HAM, Liao [62] suggested to update the initial guess at each iteration by using the solution of the previous step as the initial guess of the current step. This approach is known as iterative HAM.

In Sect. 5, both traditional HAM and iterative HAM are used to obtain the analytical solutions of static deflection of composite beams with and without elastic foundations. The efficacy of iHAM over HAM in solving beam static deflection problems is demonstrated, and then, the convergence and accuracy of the approach are investigated by comparing convergence rates and CPU time required to obtain results of specific levels of precision.

## Results and discussion

### Preliminary studies

Preliminary studies of the convergence and computational efficiency of both approaches of HAM, conventional and iterative, are performed in this Subsection. For that purpose, a cantilever laminated fibre-reinforced slender beam without elastic foundation (i.e. $k_w(x) = 0$) with boundary conditions expressed by Eq. (15.1) at $x = 0$ and Eq. (16.1) at $x = \ell$ is considered. To investigate the effect of material properties on the convergence of the solution, three types of the layup introducing different combinations of coupling terms are assumed, namely symmetric $[45_3]_s$ with bend–twist coupling, cross-ply $[0_3/90_3]$ with axial–bend coupling, and non-symmetric $[60_3/30_3]$ with bend–twist, axial–twist, and axial–bend coupling. The corresponding stiffness matrices, obtained by using closed-form expressions developed by Yu and Hodges [67], are presented in Table 1.

**Table 1:** Stiffness matrices for different types of stacking sequences

Stacking sequence	Stiffness matrix
$[45_3]_s$	$$\begin{bmatrix} 1101466 &{}\quad 0 &{} \quad 0 &{}\quad 0 \\ 0 &{}\quad 0.1764 &{}\quad -0.0591 &{}\quad 0 \\ 0 &{}\quad -0.0591 &{} \quad 0.0714 &{}\quad 0 \\ 0 &{}\quad 0 &{} \quad 0 &{}\quad 917.8885 \end{bmatrix}$$
$[0_3/90_3]$	$$\begin{bmatrix} 5481178 &{}\quad 0 &{}\quad 884.7508 &{}\quad 0 \\ 0 &{}\quad 0.0824 &{}\quad 0 &{}\quad 0 \\ 884.7508 &{}\quad 0 &{}\quad 0.2569 &{}\quad 0 \\ 0 &{}\quad 0 &{}\quad 0 &{} \quad 4567.6484 \end{bmatrix}$$
$[60_3/30_3]$	$$\begin{bmatrix} 1637714 &{}\quad 157.9162 &{}\quad -160.2034 &{}\quad 0 \\ 157.9162 &{}\quad 0.1667 &{}\quad -0.0721 &{}\quad 0 \\ -160.2034 &{}\quad -0.0721 &{}\quad 0.1009 &{}\quad 0 \\ 0 &{}\quad 0 &{}\quad 0 &{}\quad 1364.7620 \end{bmatrix}$$

The following material properties are assumed: $E_{11} = 135.64$ GPa, $E_{22} = 10.14$ GPa, $G_{12} = 5.86$ GPa, and $\nu _{12} = 0.29$. While the proposed mathematical model presented by Eq. (14) allows three types of distributed loads along the beam axis and torque, in the numerical examples only transverse load $q_z$ acting in the vertical direction is considered and used to normalise the deflections as follows:

34 $$\begin{aligned} \overline{u} = u \frac{b h E_{22}}{q_z \ell ^2}, \quad \quad {\overline{\varphi }} = \varphi \frac{b h^3 G_{12}}{q_z \ell ^3}, \quad \quad \overline{w}= w \frac{100 \, b h^{3} E_{22}}{q_{z} \ell ^{4}}. \end{aligned}$$

The following solutions of the uncoupled equations of isotropic beam are used as initial guesses:

35.1 $$\begin{aligned} u_0(x)&= \left( \ell \, x - \frac{1}{2} x^2 \right) \frac{q_x}{EA}, \end{aligned}$$

35.2 $$\begin{aligned} \varphi _0(x)&= \left( \ell \, x - \frac{1}{2} x^2 \right) \frac{q_{\varphi }}{GJ}, \end{aligned}$$

35.3 $$\begin{aligned} w_0(x)&= \left( \frac{1}{24} x^4 - \frac{\ell }{6} x^3 + \frac{\ell ^2}{4} x^2 \right) \frac{q_z}{EI_y}, \end{aligned}$$

35.4 $$\begin{aligned} v_0(x)&=\left( \frac{1}{24} x^4 - \frac{\ell }{6} x^3 + \frac{\ell ^2}{4} x^2 \right) \frac{q_y}{EI_z}. \end{aligned}$$

Before comparing the two HAM approaches, an appropriate order of deformation equation for iHAM should be established. For that purpose, iHAM results are obtained for a symmetric cantilever beam under the action of uniformly distributed load for different orders of deformation equation, namely $N = 2$, $N = 3$, and $N = 4$, using Eq. (35.1) as initial guesses and $\hbar = -1$. Using these results and those obtained from the exact solution [60], the relative error was calculated as

36 $$\begin{aligned} \text {Error} = \left| \dfrac{\text {Exact} - \text {iHAM}}{\text {Exact}} \right| \times 100 \%. \end{aligned}$$

**Fig. 2:**
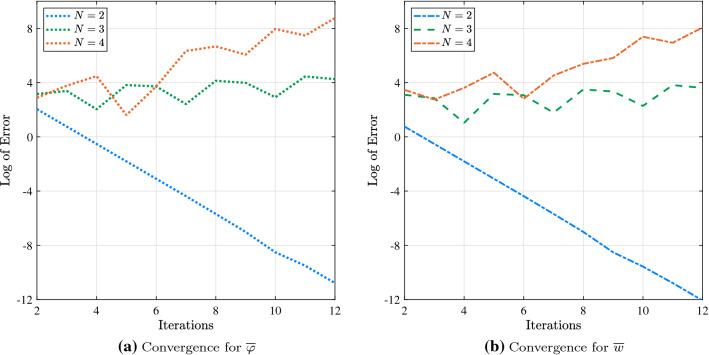
Convergence study of iHAM results for a symmetric cantilever beam under the action of uniformly distributed load for different orders of deformation equation

Figure 2 demonstrates that for both degrees of freedom available for symmetric layup, the log of error is decreasing rapidly when $N = 2$, while increasing the order of deformation equations up to $N = 3$ or $N = 4$ leads to divergent results. Thus, using the second-order deformation equation is a natural choice, and iHAM results throughout the paper are obtained based on this assumption.

**Fig. 3:**
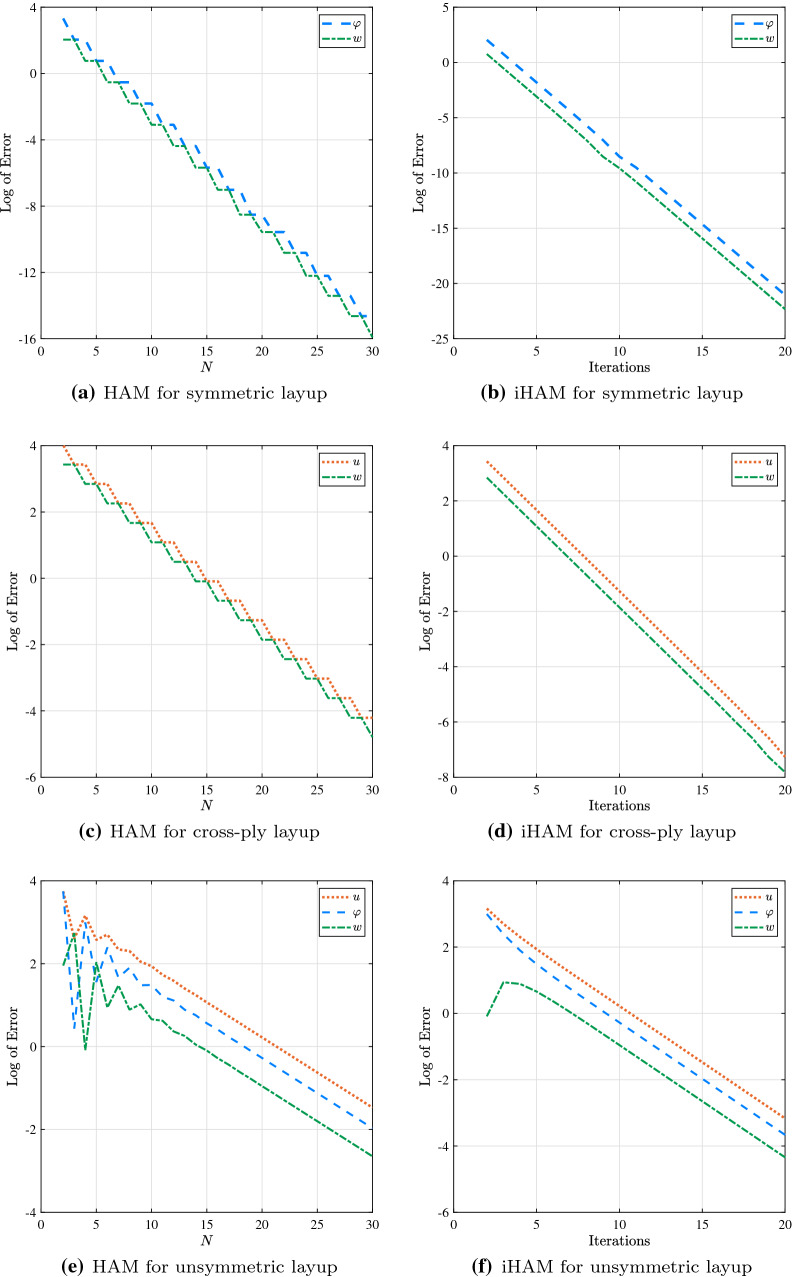
Convergence of HAM results for a cantilever beam under the action of uniformly distributed load for different types of the layup: HAM vs iHAM

With this in mind, the convergence of both algorithms of HAM can be investigated. As shown in Fig. 3, both traditional and iterative approaches provide converged results for all three types of stacking sequence. Generally, it is observed that better accuracy is obtained for symmetric and cross-ply layups compared to the non-symmetric layup which can be attributed to the occurrence of more coupling terms resulting in higher complexity. For example, the 10th-order deformation equation is sufficient to match exact results up to three decimal places in the case of the symmetric layup. In contrast, 25th-order and 35th-order deformation equations are required to attain the same level of accuracy in the cross-ply and non-symmetric layups, respectively. It is also worth noting that the convergence of iHAM results exhibits more stable behaviour compared to HAM results.

**Fig. 4:**
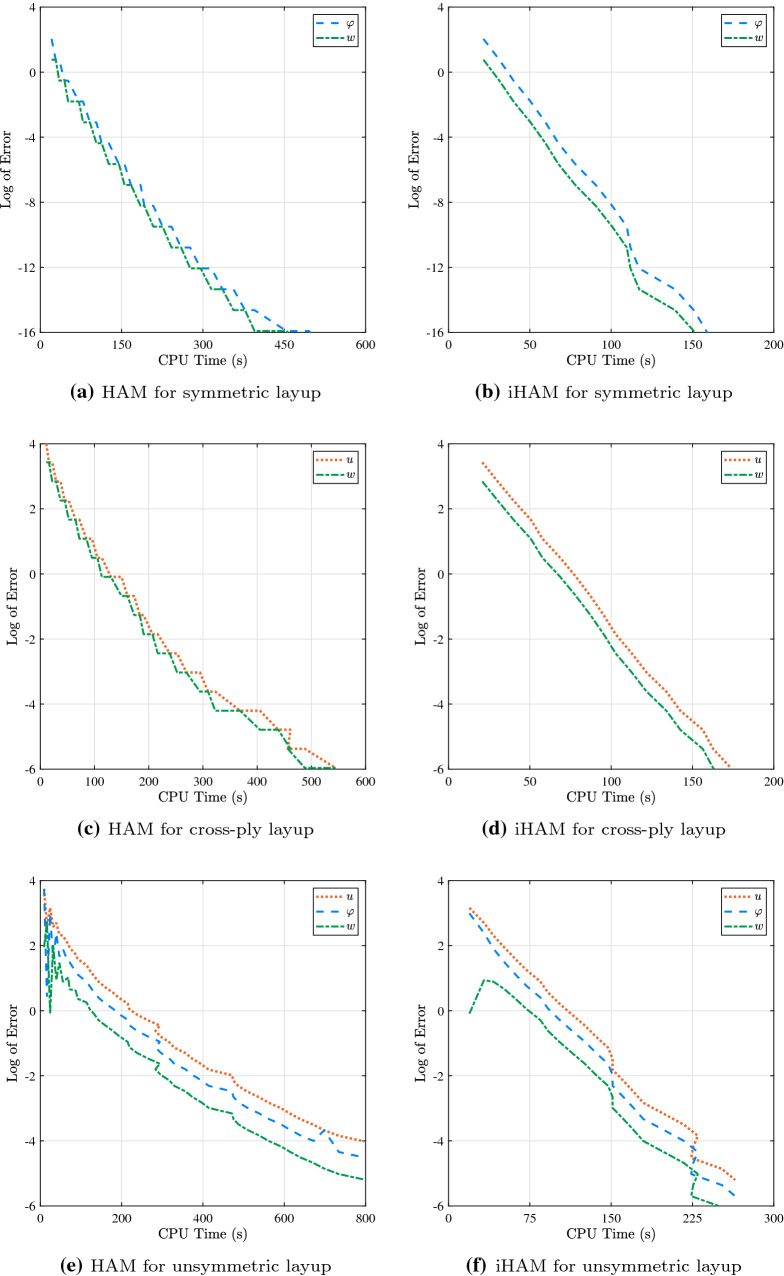
Comparison of CPU time and log of error for various types of layup for a cantilever beam under the action of uniformly distributed load: HAM vs iHAM

In order to demonstrate the computational efficiency of conventional HAM and iHAM, CPU time versus log of relative error is depicted in Fig. 4. It is worth noting that the calculations are performed using a 1.8 GHz Intel Core i5 system. Both conventional and iterative approaches allow results up to a specific degree of precision to be achieved. However, it is observed that for the same level of accuracy iHAM needs less CPU time than the traditional HAM. It is noted that iHAM converges approximately three times as fast as HAM.

**Fig. 5:**
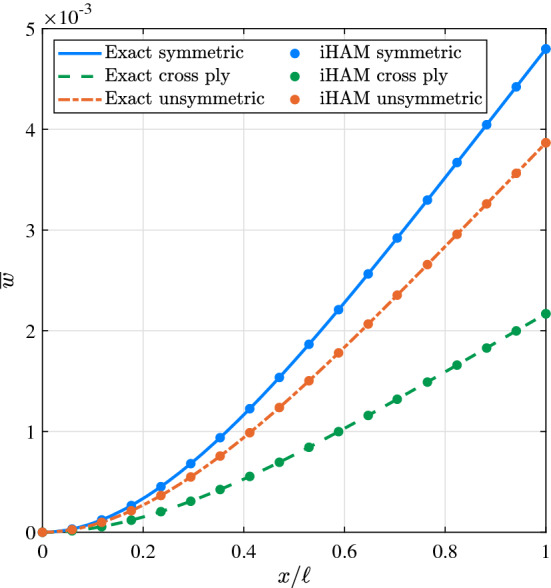
Bending $\overline{w}$ of a cantilever composite beam under the action of uniformly distributed load for different types of stacking sequences

The nondimensionalised transverse bending deflections for symmetric, cross-ply, and non-symmetric layups are shown in Fig. 5. Table 2 provides numerical results for the tip deflections of all three types of layups. It is shown that iHAM results are in good agreement with those obtained from the exact solution.

**Table 2:** Maximum deflection of a cantilever composite beam under the action of uniformly distributed load

				$[45_3]_s$		$[0_3/90_3]$		$[60_3/30_3]$
Exact [60]		$\overline{u}$		–		2.988876e+01		2.037204e+01
		${\overline{\varphi }}$		1.238568e-03		–		1.114058e-03
		$\overline{w}$		4.797695e-03		2.169912e-03		3.865681e-03
iHAM		$\overline{u}$		–		2.988072e+01		2.036345e+01
		${\overline{\varphi }}$		1.238565e-03		–		1.113772e-03
		$\overline{w}$		4.797691e-03		2.169588e-03		3.866183e-03

### Verification studies

In this Subsection, the validity and potential of the Homotopy Analysis Method in solving the static deflection problem of beams resting on elastic foundations are verified. Since numerical results for bending of fully coupled composite beams on variable elastic foundations are not available in the literature, static deflection analysis of homogeneous isotropic and composite beams on constant stiffness elastic foundations considered in several well-known studies is performed, and the results are compared with analytical and numerical results existing in the literature.

First, values of mid-span deflection of a uniformly loaded isotropic beam with clamped–clamped and simply supported–simply supported boundary conditions obtained from the present HAM and iHAM solutions, exact analytical solution [61], analytical solution based on Green’s functions [68], and the DQM solution [69] are presented in Table 3. For convenience, the mid-span transverse deflection and Winkler elastic foundation parameter are normalised as follows:

37 $$\begin{aligned} \overline{w} = \frac{w EI_y}{q \ell ^4}, \quad \overline{k}_w = \frac{k_w \ell ^4}{EI_y}, \end{aligned}$$

where $EI_y$ is the flexural rigidity and the length-to-thickness ratio $\ell /h = 120$. The analysis has been performed for three different values of $\overline{k}_w$ resulting in an elastic foundation of different stiffnesses, namely $\overline{k}_w = 0$ meaning there is no elastic foundation; $\overline{k}_w = 10$ and $\overline{k}_w = 100$ describing stiffer foundations. The results obtained from different theories for different values of the elastic foundation parameter $\overline{k}_w$ are in close agreement.

**Table 3:** Mid-span deflection $\overline{w} \times 10^{-2}$ of a uniformly loaded isotropic beam for different boundary conditions

$\overline{k}_w$	Present (HAM)	Present (iHAM)	Present (CCM)	Exact [61]	DQM [69]	Analytical [68]
*Clamped—clamped*						
0	0.260417	0.260417	0.260417	0.260417	0.26064	0.2616
10	0.255256	0.255256	0.255256	0.255256	0.25547	0.2565
100	0.216547	0.216547	0.216547	0.216547	0.21670	0.2174
*Simply supported—simply supported*						
0	1.302083	1.302083	1.302083	1.302083	1.302290	1.3033
10	1.180396	1.180396	1.180396	1.180396	1.180567	1.1814
100	0.640019	0.640019	0.640020	0.640020	0.640074	0.6403

It is worth mentioning that HAM results were obtained using $N = 20$, while iHAM results were obtained using second-order deformation equations with 8 iterations. In both approaches, $\hbar = - 1$ was assumed.

Next, nondimensional mid-span deflection of a uniformly loaded clamped laminated composite beam resting on a constant stiffness elastic foundation is obtained considering symmetric $[45_3]_s$, cross-ply $[0_3/90_3]$, and unsymmetric $[60_3/30_3]$ stacking sequences. Results obtained based on the conventional and iterative algorithms of HAM, CCM, and from the exact solution [61] are given in Table 4 for normalised Winkler elastic foundation parameter $\overline{k}_w$ taking values 0, 10, and 100. It is noted that $\hbar _i = -1$, $i = u, \varphi , w, v$, was used for both HAM approaches. The order of deformation equation required to obtain conventional HAM results of reasonable accuracy for a beam with symmetric stacking sequence was $N = 12$. With the increasing complexity of the combination of coupling terms in cross-ply and unsymmetric layups, the order of deformation equation was increased up to $N = 20$ and $N = 26$, respectively. Similarly, the number of iterations in iHAM was growing from 6 for the case of symmetric layup up to 10 and 12 for cross-ply and unsymmetric cases, respectively. It is worth mentioning that for iHAM the length of the homotopy approximations increases at each iteration exponentially which may affect the computational efficiency of the method. To address this issue, Liao [62] proposed that an approximate solution could be truncated. However, this strategy was not applied in the current work since CPU times required were acceptable. The results obtained from the exact, analytical, and numerical solutions are in good agreement.

**Table 4:** Mid-span deflection $\overline{w}$ of a uniformly loaded clamped composite beam for different stacking sequences

$\overline{k}_w$		Present (HAM)		Present (iHAM)		Present (CCM)		Exact [61]
$$[45_3]_s$$								
0		2.156656e-02		2.156656e-02		2.157635e-02		2.157635e-02
10		2.150900e-02		2.150900e-02		2.150974e-02		2.150974e-02
100		2.092799e-02		2.092799e-02		2.092803e-02		2.092803e-02
$$[0_3/90_3]$$								
0		9.734561e-03		9.734561e-03		9.762019e-03		9.762019e-03
10		9.733223e-03		9.733223e-03		9.748361e-03		9.748361e-03
100		9.601619e-03		9.601619e-03		9.627112e-03		9.627112e-03
$$[60_3/30_3]$$								
0		1.735452e-02		1.735452e-02		1.738319e-02		1.738319e-02
10		1.731068e-02		1.731068e-02		1.734039e-02		1.733993e-02
100		1.692484e-02		1.692484e-02		1.695993e-02		1.695993e-02

**Table 5:** Effect of Winkler elastic coefficient $\overline{k}_0$ on deflections of a simply supported composite beam on an elastic foundation under the action of uniformly distributed load for different types of stacking sequence

		$$\overline{k}_0$$			
		0	10	50	100
$$[45_3]_s$$					
iHAM	$\overline{\varphi }$	1.287113e-02	1.150044e-02	7.975712e-03	5.654636e-03
	$\overline{w}$	8.399915e-02	7.563204e-02	5.402032e-02	3.977959e-02
CCM	$\overline{\varphi }$	1.287158e-02	1.150156e-02	7.972653e-03	5.650985e-03
	$\overline{w}$	8.399936e-02	7.562581e-02	5.402717e-02	3.976579e-02
$$[0_3/90_3]$$					
iHAM	$\overline{u}$	8.620628e+00	7.586517e+00	5.055788e+00	3.495280e+00
	$\overline{w}$	2.710536e-02	2.405213e-02	1.650481e-02	1.185757e-02
CCM	$\overline{u}$	8.628143e+00	7.589709e+00	5.055090e+00	3.490893e+00
	$\overline{w}$	2.711009e-02	2.403250e-02	1.650695e-02	1.183994e-02
$$[60_3/30_3]$$					
iHAM	$\overline{u}$	5.876021e+00	5.214914e+00	3.597157e+00	2.532737e+00
	$\overline{\varphi }$	1.157179e-02	1.028300e-02	7.081661e-03	4.987283e-03
	$\overline{w}$	6.159597e-02	5.539938e-02	3.910800e-02	2.862306e-02
CCM	$\overline{u}$	5.880901e+00	5.236643e+00	3.597156e+00	2.534189e+00
	$\overline{\varphi }$	1.157763e-02	1.030929e-02	7.081660e-03	4.989015e-03
	$\overline{w}$	6.155864e-02	5.523198e-02	3.910800e-02	2.861297e-02

**Fig. 6:**
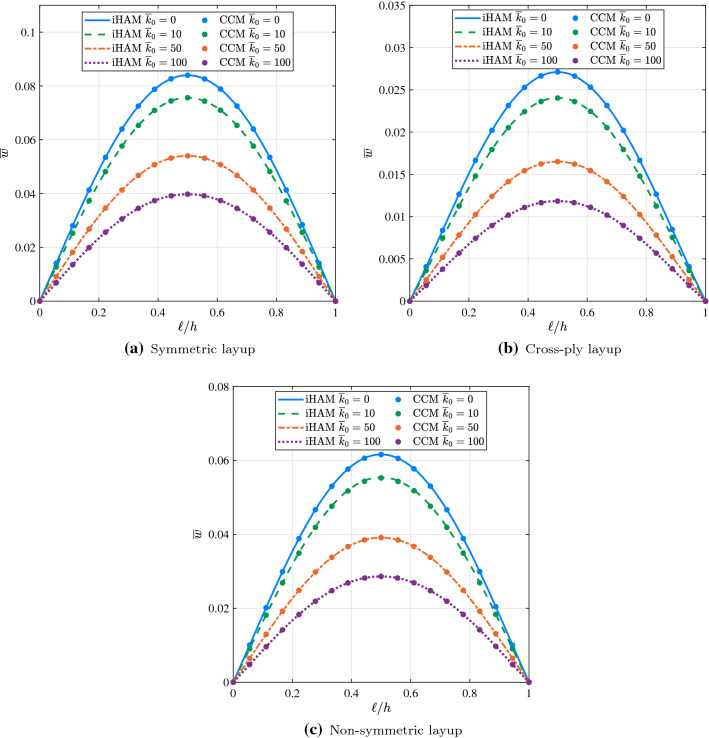
Effect of $k_0$ on the deflection $\overline{w}$ of a simply supported composite beam on linear elastic foundation under the action of uniformly distributed load for different types of the layup

### Composite beam on variable stiffness elastic foundation

In the previous Subsections, the efficiency of HAM in solving static problems of beams resting on elastic foundations was demonstrated. In addition, the superiority of an iterative approach over a conventional one was shown. Based on these results, in this Subsection iHAM analysis is extended to a composite beam resting on variable stiffness Winkler elastic foundation. The stiffness of this foundation varies linearly according to the rule described by Eq. (17) and is normalised as

38 $$\begin{aligned} \overline{k}_0 = k_0 \frac{\ell ^4}{EI_y}. \end{aligned}$$

Symmetric simply supported boundary conditions of a beam are expressed by Eqs. (15.1)–(15.4) and (16.5)–(16.6) at both $x = 0$ and $x = \ell$. With respect to these boundary conditions, the solutions of uncoupled equations of isotropic beams are obtained and used as initial guesses,

39.1 $$\begin{aligned} u_0(x)&= \frac{1}{2} \left( \ell - x^2 \right) \frac{q_x}{EA}, \end{aligned}$$

39.2 $$\begin{aligned} \varphi _0(x)&= \frac{1}{2} \left( \ell - x^2 \right) \frac{q_{\varphi }}{GJ}, \end{aligned}$$

39.3 $$\begin{aligned} w_0(x)&= \left( \frac{1}{24} x^4 - \frac{\ell }{12} x^3 + \frac{\ell ^3}{24} x \right) \frac{q_z}{EI_y}, \end{aligned}$$

39.4 $$\begin{aligned} v_0(x)&= \left( \frac{1}{24} x^4 - \frac{\ell }{12} x^3 + \frac{\ell ^3}{24} x \right) \frac{q_y}{EI_z}. \end{aligned}$$

To study the effect of Winkler elastic coefficient $k_0$ on the static behaviour of a composite beam, the deflections of a beam with various stacking sequences were analysed assuming the linear variation parameter $a = 0$ while $k_0$ was allowed to vary from 0 to 100, noting $k_0 = 0$ describes the beam without elastic foundation, whereas $k_0 \ne 0$ corresponds to a Winkler elastic foundation of constant stiffness. Table 5 gives a comparison between the analytical iHAM results and numerical approximations obtained by CCM. The nondimensionalised values of deflections obtained from both methods are in good agreement. It is worth noting that to obtain iHAM results for all values of $k_0$, auxiliary parameters $\hbar _u$, $\hbar _{\varphi }$, and $\hbar _v$ equal to $-1$ were used, while $\hbar _w$ was assumed to be equal to $-1$ for $k_0 = 0$ and $k_0 = 10$, and to $-1/2$ for $k_0 = 50$ and $k_0 = 100$, respectively. It is worth mentioning that there are different approaches for finding appropriate values of $\hbar$, for example, plotting the so-called $\hbar$-curve which allows the valid region of the convergence control parameter to be identified, or making use of the traditional square residual error employing integration or corresponding discrete form (for more details see [62]). However, for complicated coupled problems plotting $\hbar$-curves may be computationally inefficient, while the exact integration approach involved in the square residual error may not be possible or computational cost of its discrete form may not be acceptable. Thus, in the current paper the trial-and-error approach is used to find the appropriate convergence control parameter $\hbar$. Also it should be noted that more iterations of iHAM are required as the value of $k_0$ increases. The deformed configurations of the beam for all three types of the stacking sequence for different values of $k_0$ are depicted in Fig. 6. It is observed that the level of deformation depends on the type of the layup, consistently increasing from symmetric to non-symmetric and then to cross-ply stacking sequences. Additionally, the inverse relationship between the value of $k_0$ and the amplitude of the beam deflection can be observed.

**Table 6:** Values of auxiliary parameters for different types of stacking sequence and different values of linear variation parameter *a*

*a*		Iterations		Auxiliary parameter						
				$\hbar _u$		$\hbar _{\varphi }$		$\hbar _w$		$\hbar _v$
$$[45_3]_s$$										
200		16		−1		−1		−1/15		−1
400		24		−1		−1/3		−1/25		−1
600		28		−1		−1/3		−1/25		−1
$$[0_3/90_3]$$										
200		24		−1/2		−1		−1/10		−1
400		28		−1/3		−1		−1/20		−1
600		36		−1/3		−1		−1/30		−1
$$[60_3/30_3]$$										
200		18		−1/2		−1		−1/15		−1
400		22		−1/2		−1		−1/30		−1
600		28		−1/4		−1		−1/45		−1

**Table 7:** Effect of nonlinear coefficient *a* on deflections of simply supported composite beam on elastic foundation under the action of uniformly distributed load for different types of stacking sequence

		*a*		
		200	400	600
$$[45_3]_s$$				
iHAM	$\overline{\varphi }$	3.219215e-03	2.677981e-03	2.323150e-03
	$\overline{w}$	7.544566e-03	4.283861e-03	3.073918e-03
CCM	$\overline{\varphi }$	3.210367e-03	2.674014e-03	2.334200e-03
	$\overline{w}$	7.523155e-03	4.264276e-03	3.068222e-03
$$[0_3/90_3]$$				
iHAM	$\overline{u}$	1.703134e+00	1.300587e+00	1.101951e+00
	$\overline{w}$	2.198838e-03	1.249605e-03	9.011295e-04
CCM	$\overline{u}$	1.732801e+00	1.389832e+00	1.195724e+00
	$\overline{w}$	2.201209e-03	1.260207e-03	9.121786e-04
$$[60_3/30_3]$$				
iHAM	$\overline{u}$	1.375694e+00	1.142247e+00	9.999858e-01
	$\overline{\varphi }$	2.708634e-03	2.246248e-03	1.957097e-03
	$\overline{w}$	5.400966e-03	3.095841e-03	2.243746e-03
CCM	$\overline{u}$	1.390488e+00	1.145358e+00	9.954423e-01
	$\overline{\varphi }$	2.737429e-03	2.254846e-03	1.959710e-03
	$\overline{w}$	5.393207e-03	3.066044e-03	2.209968e-03

**Fig. 7:**
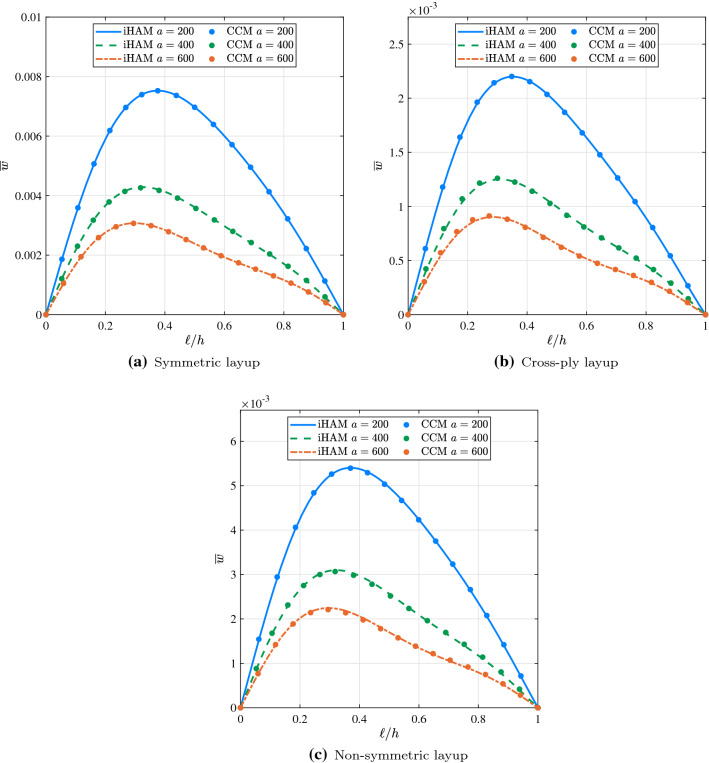
Effect of *a* on the deflection $\overline{w}$ of a simply supported composite beam on linear elastic foundation under the action of uniformly distributed load for different types of the layup

To investigate the effect of the linear variation parameter *a* on the deflection of the composite beam resting on a variable stiffness elastic foundation, Winkler elastic coefficient $k_0$ was assumed to be 10 while *a* was allowed to vary from 200 to 600. Using iHAM and CCM, the nondimensional deflections were calculated for each problem and are reported in Table 7. The results obtained from both methods are generally in good agreement. It is worth noting that the level of accuracy of iHAM results presented in Table 7 is significantly influenced by the choice of auxiliary parameters, and more iterations are required to obtain the accurate iHAM results as the value of the linear variation parameter *a* increases. For the current example values of $\hbar _i$, $i=u, \varphi , w, v$, corresponding to different degrees of freedom, are presented in Table 6. The deformed configurations of simply supported composite beams resting on linearly variable elastic foundations for different types of the layup are shown in Fig. 7. It is shown that the amplitude of the deflection depends inversely on the value of the coefficient of the variable term, namely linear variation parameter *a*. In addition, the value of this parameter affects the symmetry of the beam deformation, meaning the higher the value of *a* the more asymmetric the shape.

## Conclusions

An explicit analysis of the static deflection of composite beams resting on variable stiffness elastic foundations subject to uniformly distributed loads described by a non-homogeneous system of coupled differential equations with the combination of constant and variable coefficients has been performed by means of the Homotopy Analysis Method. Two HAM algorithms, i.e. conventional and iterative, have been implemented, and the applicability and accuracy of the method were investigated. The main difference between the two algorithms is in the method of achieving more accurate results: in conventional HAM the order of deformation equation increases for this purpose, while in iHAM the initial guess is updated at each iteration. Numerical simulations of the static deflection of composite beams with various coupling terms without elastic foundations were performed to demonstrate the superiority of the iterative approach over the conventional type by the comparison of their convergence rates. It was shown that iHAM converges more rapidly (up to three times faster than HAM) and exhibits more stable behaviour. Comparison of the results obtained by HAM and iHAM with those available in the literature shows the viability of using both approaches in the static analysis of beams resting on elastic foundations. Finally, iHAM was applied to the static analysis of composite beams resting on variable stiffness Winkler elastic foundations with different values of elastic foundation coefficient. The ensuing analytical results were compared against those obtained from the Chebyshev Collocation Method, and excellent agreement between them was observed. It was also noted that the convergence of HAM results correlates with the complexity of combination of coupling terms provided by different stacking sequences noting the results for symmetric and cross-ply layups are more accurate than for the non-symmetric layup. Values of the elastic foundation coefficient are another factor significantly influencing the computational performance of the method. It was observed that higher values of these coefficients require a larger number of iterations in obtaining iHAM results of reasonable accuracy. Thus, it can be concluded that the iterative Homotopy Analysis Method is a convenient and efficient method for the static analysis of composite beams resting on variable stiffness elastic foundations.
